# Silica Nanoparticle-Infused Omniphobic Polyurethane Foam with Bacterial Anti-Adhesion and Antifouling Properties for Hygiene Purposes

**DOI:** 10.3390/nano13142035

**Published:** 2023-07-09

**Authors:** Dongik Cho, Jun Kyun Oh

**Affiliations:** Department of Polymer Science and Engineering, Dankook University, 152 Jukjeon-ro, Suji-gu, Yongin-si 16890, Gyeonggi-do, Republic of Korea; dcdk0227@dankook.ac.kr

**Keywords:** omniphobic, polyurethane foam, bacteria, antifouling, hygiene

## Abstract

In this study, a method for preventing cross-infection through the surface coating treatment of polyurethane (PU) foam using functionalized silica nanoparticles was developed. Experimental results confirmed that the fabricated PU foam exhibited omniphobic characteristics, demonstrating strong resistance to both polar and nonpolar contaminants. Additionally, quantitative analysis using the pour plate method and direct counting with a scanning electron microscope determined that the treated material exhibited anti-adhesion properties against bacteria. The fabricated PU foam also demonstrated a high level of resistance to the absorption of liquids commonly found in medical facilities, including blood, 0.9% sodium chloride solution, and 50% glycerol. Mechanical durability and stability were verified through repeated compression tests and chemical leaching tests, respectively. The proposed coated PU foam is highly effective at preventing fouling from polar and nonpolar fluids as well as bacteria, making it well-suited for use in a range of fields requiring strict hygiene standards, including the medical, food, and environmental industries.

## 1. Introduction

Recently, with the emergence of the highly contagious novel virus infection, concerns have been raised about infectious diseases around the world. Cross-contamination, in which pathogenic bacteria are transferred through contact with an infected person or material, is a major cause of the spread of such infectious diseases. Medical facilities try to prevent cross-contamination through the use of disposable items as well as strict disinfection and cleaning management. For example, the hospital disposables market is growing, with an average annual growth rate of 9.4% expected until 2026. However, along with the increased use of disposables, the waste generated is also increasing. Medical facilities in the United States generate 4 million tons of waste each year [1]. The cost of disposing this waste accounts for about 20% of the cost of hospital environmental services; thus, not only environmental pollution caused by the waste but also economic costs are becoming a problem [2]. In addition to the use of disposable items, cross-contamination is prevented by methods such as disinfection and cleaning, but for hospital mattresses, bacterial contamination is a problem, even after disinfection. According to French et al., the mattresses used by patients infected with methicillin-resistant *Staphylococcus aureus* (MRSA) remained contaminated with MRSA even after cleaning, and 43% of uninfected patients subsequently placed in these beds became infected with MRSA [3]. Furthermore, based on research conducted by Sharma et al., sulfadiazine antibiotics, which are used as therapeutic agents to inhibit bacterial growth, have certain limitations in their accurate detection methods. Moreover, they not only have the potential to impact the ecosystem but also pose a risk to the human body [4]. These cases suggest that disinfection and cleaning performed as a method for preventing cross-contamination cannot be a fundamental solution.

As patients spend the most time in a medical facility in the hospital bed, the materials most likely to cause infection or the transfer of pathogenic bacteria include mattresses, pillows, and filling materials on which patients lie [5,6]. Polyurethane (PU) foams, which are one of the many types of PU, are mainly used for these materials. PU can be used as a foam, coating, elastomer, or adhesive, depending on the ratio of polyol and isocyanate, owing to its excellent processability and easily controlled physical properties. Among the many forms of PU, PU foams are the most commonly used owing to their high surface area-to-volume ratio, excellent thermal properties, and elasticity [7]. PU foams can be divided into open-cell foams and closed-cell foams, depending on the internal structure [8]. A higher open-cell ratio affects softness and volume shrinkage, and most mattresses (almost 100%) have an open-cell structure that makes them easy to clean [9]. The PU foams of hospital mattresses with a very high percentage of an open-cell structure have a porous network structure, and because of this, they have a very high absorbency for various liquids such as water and oil compared to PU foam with a closed-cell structure. Therefore, for mattresses used by a large number of patients, oil or body fluids generated from the human body can easily penetrate into the cells of the PU foam. This creates an environment conducive to the propagation of biological contaminants such as bacteria, increasing the risk of healthcare-associated infections due to cross-contamination and bacterial transmission [10].

To prevent cross-contamination and bacterial transfer by PU foam used as a mattress material, studies on surface antimicrobial coatings have been conducted to analyze the antibacterial properties of PU foam. A number of studies used antimicrobial agents [11]. For example, Dagostin et al. reported the antimicrobial effect of PU foam treated by adding 0.5 wt% of zinc pyrithione, an antimicrobial agent [12]. Ashjari et al. prepared an antimicrobial mattress in which the growth of bacteria was inhibited by adding CuO to PU foam prepared based on starch [13]. Furthermore, Demirci et al. prepared an open-cell antimicrobial PU foam using quaternary ammonium compounds that had an antimicrobial effect with cationic biocides [14]. Additionally, according to Sienkiewicz et al., bio-based compounds are utilized to provide effective antibacterial and antifungal effects in construction, which is another field where polyurethane foam finds widespread use [15].

The PU foams containing the reported antimicrobial agents can prevent cross-infection by killing bacteria and preventing growth, but the antimicrobial effect decreases over time because of the exhaustion of the antimicrobial agent. The resistance of bacteria to antimicrobial agents may also increase, and when bacterial corpses due to antimicrobial agents form a biofilm on the surface, the antibacterial effect is rapidly reduced, which is inefficient in the long term. A method of preventing cross-infection and bacterial transfer that overcomes these disadvantages is to prepare an anti-adhesion or antifouling surface that prevents bacterial adhesion [16]. The anti-adhesion surface does not kill or inhibit the growth of bacteria but reduces the surface energy of the material to prevent the adhesion of bacteria, thereby reducing the possibility of antimicrobial resistance or biofilm formation [17]. Therefore, to prevent the adhesion of polar contaminants such as bacteria and fungi, as well as nonpolar contaminants (oil and lipid components), an omniphobic surface with repellency to both polar and nonpolar substances is essential.

In contrast to superhydrophobic surfaces with a limited antifouling effect that can only prevent the adhesion of bacteria and polar contaminants, an omniphobic material surface has the advantage of having antifouling effects against both polar and nonpolar contaminants [18]. Omniphobic properties can be determined based on the contact angle between the liquid and the surface; if the contact angles of the surface with water and oil are both greater than 150°, it is considered an omniphobic surface. A lot of research on omniphobic surfaces has been conducted. For example, Pendurthi et al. formed a surface nanomorphology with a re-entrant structure by using a CO_2_ laser engraver on the surfaces of stainless steel, aluminum, and glass to create a surface that is repellent to water and oil [19]. Li et al. prepared an omniphobic membrane that can be used as a distiller filter by coating a fluorocarbon surfactant, a fluorinated alkyl silane, and silica (SiO_2_) nanoparticles on a polyvinylidene fluoride (PVDF) membrane using a spray coating method [20]. Zhang et al. created omniphobic surfaces by slippery liquid-infused porous surfaces (SLIPSs) with liquid containing ZnO, Co_3_O_4_, and SiO_2_ [21]. Wu et al. fabricated a nanofibrous membrane on a poly(vinylidene fluoride)-*co*-hexafluoropropylene (PVDF-HFP) membrane using the electrospinning method and then fluorinated the surface with a vapor deposition method to impart omniphobic properties [22]. It is difficult to apply this method to products with a short replacement period, such as mattresses, because the control of accurate surface topography is complicated and expensive [23]. In addition, because there is no mention of an experiment confirming bacterial anti-adhesion, a follow-up study on this is necessary. Research confirming the antibacterial properties of PU foam with an omniphobic surface related to the medical field is also required.

In this study, inexpensive reagents and dip-coating methods that could be scalable were applied, with the consideration of inexpensive reagents and a mass production capability for the manufacture of PU foam with omniphobic properties. The aim was an antifouling effect against bacteria as well as polar and nonpolar pollutants that commonly occur in medical environments. To confirm the surface properties of PU foam coated with fluorinated silane-coated silica nanoparticles (FSCSNs), Fourier transform infrared (FTIR) spectroscopy, contact angle measurement, and scanning electron microscopy (SEM) were used. Experiments were conducted to confirm the antibacterial properties of PU foam coated with FSCSNs, in which the Gram-negative bacteria *E. coli* O157:H7 and Gram-positive bacteria *S. epidermidis* were inoculated for the analysis. To confirm the anti-absorption properties of PU foam coated with FSCSNs for polar and nonpolar contaminants, polar liquids (sterile deionized (DI) water, 0.9% sodium chloride solution, and blood) and nonpolar liquids (hexadecane, 50% glycerol solution, and squalene) commonly found in medical facilities were used. A repeated compression test was performed using a counterweight to confirm the durability of the PU foam coated with FSCSNs. Finally, a chemical leaching test was conducted to evaluate the chemical stability. Considering that PU foam is a material that is in contact with the patient’s body for the longest time in medical facilities and is highly likely to be exposed to vectors of infectious diseases, the fabricated PU foam is expected to be used as a hospital material that prevents healthcare-associated infections due to cross-infection and bacterial transfer through omniphobic properties achieved using a simple dip-coating method.

## 2. Materials and Methods

### 2.1. Preparation of PU Foam Samples

The polyurethane (PU) foam (density of 30.3 kg/m^2^, tensile strength of 1.25 kg/m^2^, elongation of 130%) used in the experiment was purchased from Kumkang Urethan (Namyangju-si, Republic of Korea). To compare the anti-adhesion effect, untreated PU foam cubes measuring 2 cm × 2 cm × 2 cm were cut, soaked in ethanol (95%, Daejung Chemicals, Siheung-si, Republic of Korea) for 5 min, and dried in an oven at 60 °C for 20 min.

### 2.2. Fluorinated Silica Nanoparticles Suspension

First, 300 mg of silica (SiO_2_; Sigma-Aldrich Co., St. Louis, MO, USA) nanoparticles with an average diameter of ca. 20 nm were added to 50 mL hexane (Daejung Chemicals, Siheung-si, Republic of Korea) and suspended for 20 min with a probe-type ultrasonicator. Then, 5 mM of trichloro(*1H,1H,2H,2H*-heptadecafluorodecyl)silane (HFTCS, Tokyo Chemical Industry, Tokyo, Japan) was added to the suspension and suspended for another 20 min to obtain FSCSNs. The FSCSNs suspension was left at room temperature for 2 h to sufficiently react the silica nanoparticles with the silane.

### 2.3. Dip-Coating

The dip-coating method was used to coat FSCSNs on PU foam. PU foam cut into 2 cm × 2 cm × 2 cm cubes was dipped in the FSCSNs suspension. Dipping in the suspension for 30 s followed by drying at room temperature for 30 s was repeated five times. The coating was performed at a speed of 0.5 cm/s so that the FSCSNs were uniformly coated. Finally, the PU foam coated with FSCSNs was dried at room temperature for 24 h to obtain FSCSNs-coated PU foam.

### 2.4. Surface Characterization

To confirm the chemical interaction and trifluoromethyl (−CF_3_) functional groups between HFTCS functionalized silica nanoparticles inside and outside the coated PU foam, we used FTIR spectroscopy. FTIR spectra were measured using a spectrometer (Nicolet iS10, Thermo Fisher Scientific, Waltham, MA, USA) and analyzed using OMNIC software (Thermo Fisher Scientific, Waltham, MA, USA).

To measure the wetting characteristics, the static contact angle of untreated (bare) PU foam and coated PU foam was measured using the sessile drop technique. The experiment involved dropping droplets of water and hexadecane of the same volume (5 μL) onto the foam surface five times at room temperature and then determining the average value from the measurements. Contact angles were analyzed using ImageJ software (National Institutes of Health, Bethesda, MD, USA) via the low-bond axisymmetric drop shape analysis (LBADSA) plugin [24].

Finally, SEM (S-4700s; Hitachi, Tokyo, Japan) was used to check the surface morphology of the coated PU foam. To enable the conductivity of the sample during the SEM measurement, a layer of platinum with a thickness of 20 nm was applied prior to the SEM analysis. The SEM was operated with a voltage of 20 kV and a current of 10 μA.

### 2.5. Bacterial Cultures

In this study, two types of bacteria were used: Gram-negative *Escherichia coli* O157:H7 (ATCC 25922) and Gram-positive *Staphylococcus epidermidis* (ATCC 12228). *E. coli* O157:H7 and *S. epidermidis* were transferred from tryptic soy agar slants (TSA; Becton, Dickinson and Co., Franklin Lakes, NJ, USA) to a culture tube containing 9.0 mL of tryptic soy broth (TSB; Becton, Dickinson and Co., Franklin Lakes, NJ, USA) using a loopful (10 μL) and cultured. The tubes of all strains were incubated aerobically at 37 °C for 24 h without shaking. A second transfer was conducted by transferring a loopful culture to a fresh TSB culture medium and then incubating under the same conditions. The experiment utilized final concentrations of the two bacteria ranging from 8.8 to 9.2 log CFU/mL.

### 2.6. Bacterial Adhesion Assay

To evaluate whether the anti-adhesion effect of the foam persists even after prolonged exposure to bacteria, bare and FSCSNs-coated PU foam samples were immersed in 9 mL of a bacterial suspension at room temperature for 1 h and 8 h. After immersion, the samples were removed from the bacterial suspension quickly and then transferred to a conical tube containing sterile DI water (9 mL) for a further bacterial adhesion assay. The bacterial adhesion assay was conducted in a suitable biological safety cabinet with sterile conditions.

The adhesion of bacteria on both the bare and FSCSNs-coated PU foam surfaces was evaluated using the pour plating method for plate counting, as well as direct counting on the PU foam surfaces using SEM. For plate counts, PU foam samples that were inoculated using conical tubes for 1 h and 8 h were each vortexed in sterile DI water for 1 min at 3000 RPM to detach bacteria from the PU foam surfaces. After that, the DI water (1 mL) containing bacteria detached from the sample was added to 0.1% (*w*/*v*) peptone water (9 mL), serial dilution was performed, and the final solution was spread on a TSA plate. The bacterial density was counted after 24 h of aerobic incubation at 37 °C and refers to the density of bacteria adhering to the PU foam surfaces. All experiments were replicated three times.

Counting the number of bacteria attached to the surface of the PU foam using SEM was performed using PU foam samples immersed in the inoculum for 1 h and 8 h. Before imaging by SEM, bacteria were inactivated using ethanol, and 20 nm of platinum coating was applied to ensure electrical conductivity for the SEM measurement. For statistical reliability, more than five areas of 100 μm × 100 μm were observed in three samples of the same type of PU foam. SEM micrographs were analyzed using ImageJ to quantify the attachment of *E. coli* O157:H7 and *S. epidermidis* to PU foam surfaces.

### 2.7. Absorption Capacity Test

The test method for measuring absorption capacity was based on ASTM F726-99 (i.e., the standard test method for the sorbent performance of adsorbents). First, in the nonpolar liquid absorption comparison test, 1 mL of hexadecane was added to a Petri dish, and the weighed bare PU foam and FSCSNs-coated PU foam were each immersed in hexadecane for 1 min. Subsequently, the PU foam was removed and left to dry at room temperature for 5 s. The saturated PU foam was then placed on a pre-weighed Petri dish, and the weight of the saturated PU foam was recorded by subtracting the weight of the Petri dish. The test was conducted in the same way using 50% glycerol and squalene, which are other nonpolar liquids used in hospitals. The absorption comparison tests for polar liquids (DI water, 0.9% sodium chloride solution, and blood), which are often used in hospitals, were also conducted using a similar method. The absorption capacity of the PU foam was calculated according to Equation (1), where *M_d_* is the initial dry weight of the PU foam, and *M_w_* is the weight of the saturated PU foam after absorbing the liquid. All absorption tests were performed under the same conditions at room temperature, and the average absorption capacity was calculated by repeating three times. In addition, if the result deviated from the average by 10%, a retest was conducted using a new sample.

(1) $$\mathrm{Absorption}\;\mathrm{capacity}\;\left(g/g\right)=\frac{\left(M_{w}-M_{d}\right)}{M_{d}}$$

### 2.8. Compression Test

A compression test was performed on FSCSNs-coated PU foam to confirm that the omniphobic properties were maintained even under repeated stress due to the characteristics of mattresses, which must withstand the weight of the human body for a long period. The mechanical properties were measured by repeatedly applying the same pressure in the vertical direction with a 500 g weight connected by string to the FSCSNs-coated PU foam with a size of 1.5 cm × 1.5 cm × 1.5 cm. The coated PU foam was subjected to a total of 100 compressions, while assessing its ability to repel liquids. To confirm the omniphobic properties of the foam, droplets of water and hexadecane (5 μL each) were applied five times each after every ten compressions, and the resulting contact angles were measured and averaged.

### 2.9. Chemical Leaching Test

The chemical stability of the FSCSNs-coated foam was determined by checking whether there was chemical leaching on the surface of the foam over time when the FSCSNs-coated foam was immersed in a polar or nonpolar liquid. Polar and nonpolar liquids were tested using 0.9% sodium chloride solution and 50% glycerol, liquids commonly used in hospitals. FTIR spectroscopy, with a detection limit of <1 ppm, was employed as the analysis method. The immersion condition was set to a maximum of 14 d, and the results were compared by performing analysis every 7 d.

### 2.10. Statistical Analysis

The bacterial adhesion assay results were analyzed after log-transformation. The significance level was set to *p* < 0.05 in the results of the bacterial attachment analysis. For this purpose, two-way analysis of variance with Tukey’s post hoc test was used. Statistical analyses were performed using the Analysis ToolPak in Excel (Microsoft Corp., Redmond, WA, USA) via statistical packages.

## 3. Results and Discussion

### 3.1. Characterization of FSCSNs-Coated PU Foam Surfaces

**Figure 1:**
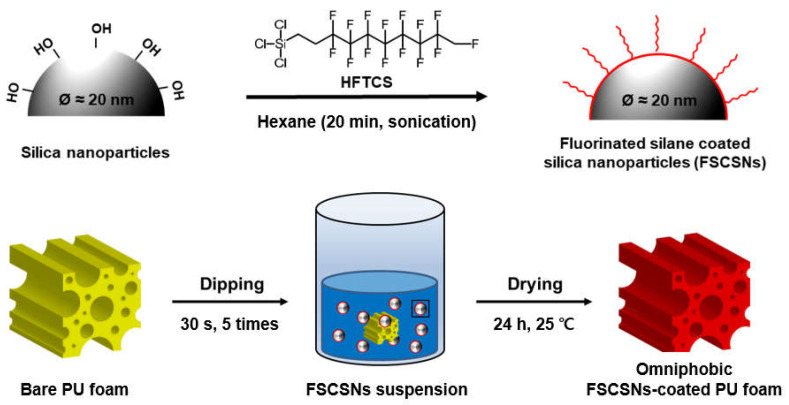
Schematic illustration of the synthesis of FSCSNs through the silanization of HFTCS and silica nanoparticles. Fabrication of FSCSNs-coated PU foam with omniphobic properties using FSCSNs suspension and the dip-coating method.

**Figure 2:**
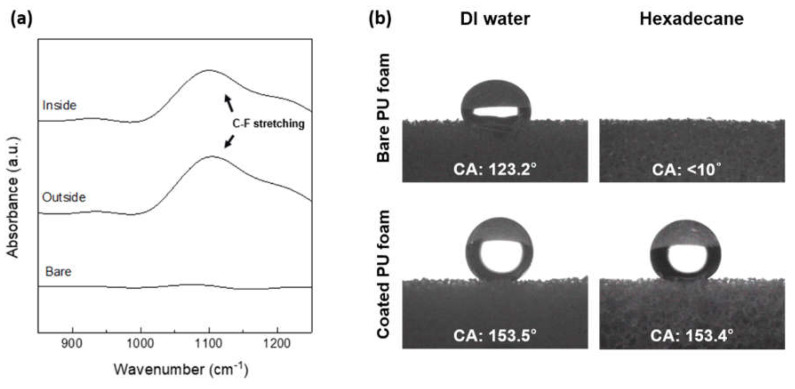
(**a**) C−F stretching region from the FTIR spectra of the inside and outside regions of the FSCSNs-coated PU foam and the bare PU foam. (**b**) Static contact angle (CA) measurement results for bare PU foam and FSCSNs-coated PU foam.

Omniphobic PU foam with both superhydrophobic and superoleophobic properties was manufactured by coating the surface of the PU foam with FSCSNs by silanization through a condensation reaction between −OH and −Cl groups on the surface of the PU foam (Figure 1). Bacterial adhesion can be affected when nano or microscopic roughness is applied to the surface through chemical or physical methods. If the surface roughness unit is larger than the size of the bacteria, the bacteria penetrate the topography, such as valleys, edges, and pits between the surface roughness, thereby increasing the contact area with enhanced bacterial adhesion on the surface as well as bacterial colonization. In addition, PU foam has a three-dimensional porous structure and a large surface area, allowing air to enter and exit smoothly and creating a suitable environment for bacteria to inhabit. Therefore, in this study, FSCSNs were coated on the porous surface of PU foam to lower the surface energy and to impart nanoscale surface roughness to achieve a high repellency against contaminants. These properties are due to HFTCS, a fluorinated silane used for surface modification, and, more specifically, due to the bond between carbon and fluorine in the chain structure of HFTCS. A carbon–fluorine (C−F) bond is a bond between carbon with an electronegativity of 2.5 and fluorine with an electronegativity of 4.0, which is a very strong bond with a large difference in electronegativity. That is, the electron density around the carbon atom is reduced and that around the fluorine atom is increased to create positive and negative charges, respectively; the charges cancel each other out so that the net charge of the perfluorinated molecule is zero. The high electronegativity of fluorine reduces the polarizability of the bond, limiting its susceptibility to van der Waals interactions (related to the magnetic susceptibility, which is the ratio of the strength of magnetization of a material to the strength of a magnetic field), leading to very low surface energies of perfluorinated materials [25]. FTIR analysis was performed to confirm the presence of trifluoromethyl functional groups on the inside and outside of the coated PU foam (Figure 2a). Although no peak was observed in the corresponding wavelength band in the bare PU foam, in the coated PU foam, a peak was observed at a wavelength of 1050 cm^−1^ for samples both inside and outside the treated PU foam, confirming that this corresponded to the C−F stretching peak. The presence of a peak at approximately 1050 cm^−1^ confirms the occurrence of C−F stretching. Furthermore, the observed peak shift of approximately 8 cm^−1^ towards lower wavelengths can be attributed to the reduction in C−F bond strength resulting from the functionalization process involving silica nanoparticles [26]. Additionally, we observed weak vibrations at 950 cm^−1^ both inside and outside the regions of the PU foam coated with FSCSNs. These vibrations were verified to be the result of an asymmetric vibration caused by Si−OH groups introduced by the presence of SNPs [27]. Therefore, it was confirmed that the porous surface of the PU foam was coated with FSCSNs [28].

The surface morphology modified by FSCSNs generated on the porous surface of PU foam and the chemical modification by HFTCS create a synergistic effect for repelling bacteria [29]. FSCSNs form a hierarchical structure on the PU foam surface. When an air layer is formed between uniformly aggregated FSCSNs and bacteria adhere to the surface, the air pocket formed between the surface and the bacteria causes an air-pocket effect, which causes the bacteria to come into contact with the surface in a nonlinear manner, eventually affecting the growth of the bacteria [30]. In this study, to compare the wettability of bare PU foam and PU foam coated with FSCSNs to that of water and oil, the static contact angle was measured for DI water and hexadecane (Figure 2b). The water contact angle of the bare PU foam was 123.2°, indicating hydrophobicity, but the water was observed to be gradually absorbed into the PU foam over time. Because the hexadecane droplet was absorbed into the PU foam as soon as it fell, the contact angle for hexadecane was indicated as <10°. In contrast, the contact angles of the PU foam coated with FSCSNs were 153.5° and 153.4° for water and hexadecane, respectively. As these contact angles indicate super water repellency and super oil repellency, respectively, the PU foam coated with FSCSNs had omniphobic characteristics due to the effect of low surface energy and nanoscale surface roughness generated by FSCSNs. The contact angle of a surface can be influenced by various physical factors, including the roughness, texture, and hierarchical structure. Simultaneously, chemical factors such as surface heterogeneity can also have a substantial impact on determining the contact angle [31,32].

**Figure 3:**
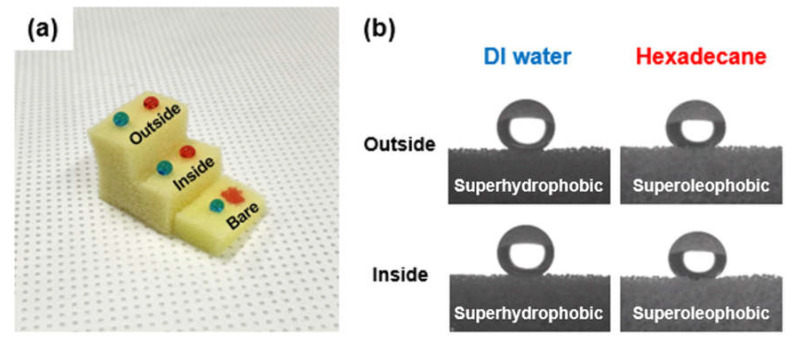
(**a**) Photographs of polar and nonpolar liquid (DI water: blue, hexadecane: red) on the inside and outside regions of FSCSNs-coated foam and bare foam. (**b**) Photographs of the static contact angle for polar and nonpolar liquids inside and outside the FSCSNs-coated foam.

Furthermore, an experiment was conducted to confirm whether both the inside and outside of the coated PU foam had omniphobic properties. Figure 3a shows photographs taken after dropping DI water (blue) and hexadecane (red) on the bare and coated PU foam samples to examine the liquid repellency of the inside and outside of the PU foam. As a result, the liquid dropped on the bare PU foam permeated inside, whereas the liquid dropped on the FSCSNs-coated PU foam maintained its spherical shape. Figure 3b shows the results of measuring the static contact angles of the inside and outside of the coated PU foam. The results confirmed that the contact angle of both the inside and outside of the coated PU foam was 150° or more, indicating that it had omniphobic properties.

**Figure 4:**
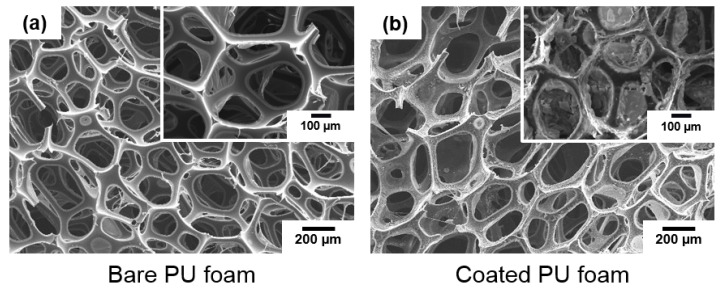
SEM micrographs of (**a**) bare and (**b**) FSCSNs-coated PU foams showing open-cell structures.

The surface morphology of PU foam with a porous structure was observed using SEM (Figure 4). On the surface of the bare PU foam, the entire surface structure had an open-cell structure, and the surface of the internal network structure was smooth. In contrast, as shown in the enlarged photograph of the surface of the PU foam coated with FSCSNs, a hierarchical structure was formed on the surface of the PU foam due to the FSCSNs. However, despite these coatings, it was confirmed that the surface morphology of the PU foam coated with FSCSNs also maintained an open-cell structure. The coating did not affect the characteristics of the mattress, as the softness, volumetric shrinkage, and shock mitigation of the mattress were determined by the open-cell structure [33].

### 3.2. Bacterial Attachment to FSCSNs-Coated PU Foam Surfaces Characterized by Plating Counting

**Figure 5:**
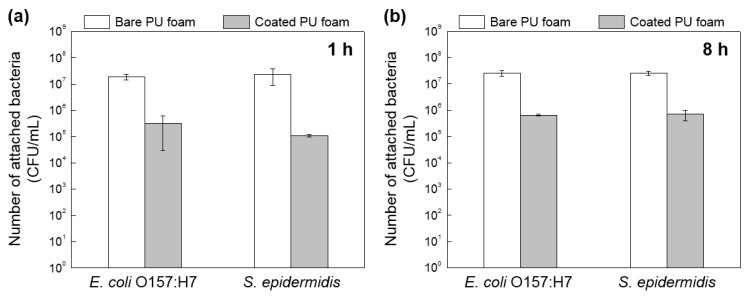
Comparison of bacterial adhesion between bare PU foam and FSCSN-coated PU foam through quantitative results after exposure to bacteria for (**a**) 1 h and (**b**) 8 h.

The bacterial adhesion behavior on the bare PU foam and the PU foam coated with FSCSNs over time was confirmed using the pour plate method. The plate counting results of the PU foam samples inoculated with (exposed to) the bacterial suspension for 1 h and 8 h are quantitatively represented graphically in Figure 5. First, the results of inoculation for 1 h, as shown in the left graph of Figure 5a, indicate that the mean population of bacteria adhered to the PU foam inoculated for 1 h in *E. coli* O157:H7 suspension was 7.3 log CFU/mL for the bare PU foam and 4.5 log CFU/mL for the PU foam coated with FSCSNs. In addition, in the results of inoculation with *S. epidermidis* shown in the right graph, the mean population of bacteria adhered to the PU foam was 7.4 log CFU/mL for the bare PU foam and 5.0 log CFU/mL for the PU foam coated with FSCSNs. In other words, as a result of immersion in the bacterial suspension for 1 h, the bacteria adhering to the coated PU foam surface were reduced by 98.9%. The trend of the 1 h adhesion test results was similar for the 8 h test. As shown in Figure 5b, the mean population of bacteria adhered to the PU foam inoculated for 8 h in *E. coli* O157:H7 suspension was 7.4 log CFU/mL for the bare PU foam and 5.8 log CFU/mL for the coated PU foam. In addition, as a result of inoculation with *S. epidermidis*, the mean population of bacteria adhered to the PU foam was 7.5 log CFU/mL for the bare PU foam and 5.7 log CFU/mL for the PU foam coated with FSCSNs. Similar to the results of inoculation for 1 h, the PU foam coated with FSCSNs showed a 97.4% decrease in the degree of adhesion after 8 h of bacterial exposure compared to the bare PU foam. This confirms the antimicrobial properties of the PU foam coated with FSCSNs; the results of the pour plate method indicated an average of a 1–2 log units decrease compared to the bare PU foam. The PU foam coated with FSCSNs could prevent the proliferation of bacteria even during the proper sleeping time (i.e., 8 h), when the body is in contact with the mattress for a long time.

### 3.3. Bacterial Attachment to FSCSNs-Coated PU Foam Surfaces Characterized by SEM

**Figure 6:**
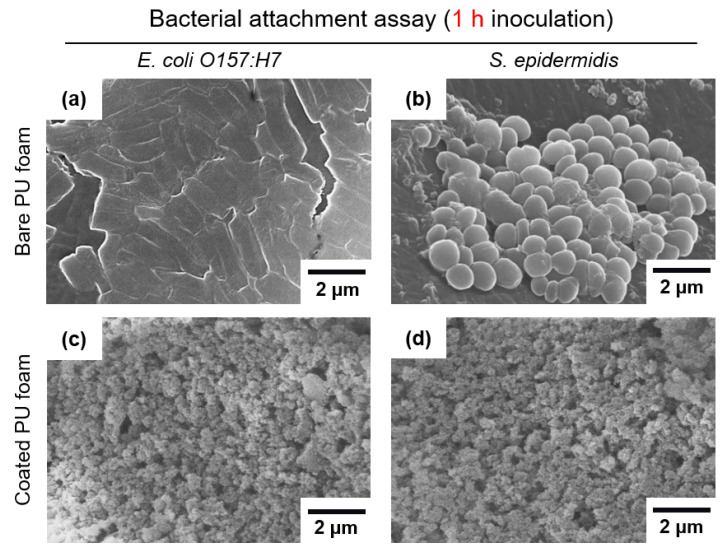
SEM micrographs of (**a**) *E. coli* O157:H7 attached to bare PU foam, (**b**) *S. epidermidis* attached to bare PU foam, (**c**) *E. coli* O157:H7 attached to FSCNSs-coated PU foam, and (**d**) *S. epidermidis* attached to FSCNSs-coated PU foam after 1 h exposure to bacteria.

In addition to the pour plate method, the number of bacteria adhered per unit area was directly counted using SEM to confirm the antimicrobial properties of the treated PU foam, and the distribution was quantified. After inoculating with the bacteria for 1 h and 8 h, the bacteria adhered to the two types of PU foam surfaces were compared. As shown in Figure 6, a large number of bacteria of both types adhered to the surface of the bare PU foam. In contrast, on the surface of the PU foam coated with FSCSNs, adhered bacteria could not be identified, and only uniform and hierarchical structures generated by FSCSNs could be identified. The number of bacteria adhered to the PU foam samples inoculated with *E. coli* O157:H7 for 1 h was 6.1 log cells/mm^2^ for the bare PU foam and 4.5 log cells/mm^2^ for the PU foam coated with FSCSNs, showing a 97.4% decrease. The number of *S. epidermidis* bacterial cells adhered to the PU foams was 6.1 log cells/mm^2^ for the bare PU foam and 4.3 log cells/mm^2^, showing a 98.3% decrease.

**Figure 7:**
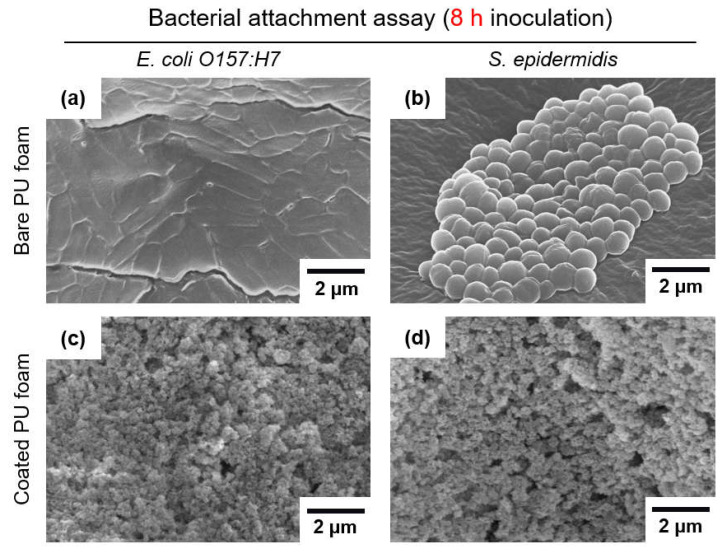
SEM micrographs of (**a**) *E. coli* O157:H7 attached to bare PU foam, (**b**) *S. epidermidis* attached to bare PU foam, (**c**) *E. coli* O157:H7 attached to FSCNSs-coated PU foam, and (**d**) *S. epidermidis* attached to FSCNSs-coated PU foam after 8 h exposure to bacteria.

After immersing the two types of PU foam in the bacterial suspension for 8 h, reflective of the sleeping time, the number of adhered bacteria was also counted. Looking at the results shown in Figure 7, the number of bacteria adhered to the PU foams inoculated with *E. coli* O157:H7 for 8 h was 6.2 log cells/mm^2^ for the bare PU foam and 4.7 log cells/mm^2^ for the coated PU foam, showing a 96.7% decrease. The number of *S. epidermidis* bacteria adhered to the PU foams was 6.1 log cells/mm^2^ for the bare PU foam and 4.5 log cells/mm^2^ for the coated PU foam, showing a 97.4% decrease. Therefore, as a result of analyzing the bacterial adhesion properties, the PU foam coated with FSCSNs had excellent anti-adhesion properties, having a significant (*p* < 0.5) reduction of 1–2 log units or more for Gram-negative and Gram-positive bacteria. As a result of indirect counting using the pour plate method described in Section 3.2 and direct counting using SEM, when omniphobic PU foam coated with FSCSNs was exposed to bacterial suspensions containing *E. coli* O157:H7 and *S. epidermidis* for 1 h and 8 h, it exhibited anti-adhesion properties against bacteria, with smaller numbers of bacteria adhered to the surface than for the bare PU foam. This phenomenon can be explained by the wetting transition of the PU foam surface from the Wenzel state to the Cassie–Baxter state caused by the FSCSN coating [34]. The transition of the PU foam surface to the Cassie–Baxter state means that the real contact area is reduced by air pockets generated when hydrophilic bacteria come into contact with the PU foam surface [35]. In other words, the anti-adhesion effect can be explained by hydrophilic and hydrophobic effects [36,37]. The surface layer of *E. coli* O157:H7 and *S. epidermidis* has a hydrophilic property at a water contact angle between 16° and 57° [38,39]. When bacteria with such a hydrophilic cell surface come into contact with a nonpolar surface, intermolecular interactions repel them, thereby hindering bacterial adhesion [40].

### 3.4. Anti-Absorption Test of FSCSNs-Coated PU Foam

**Figure 8:**
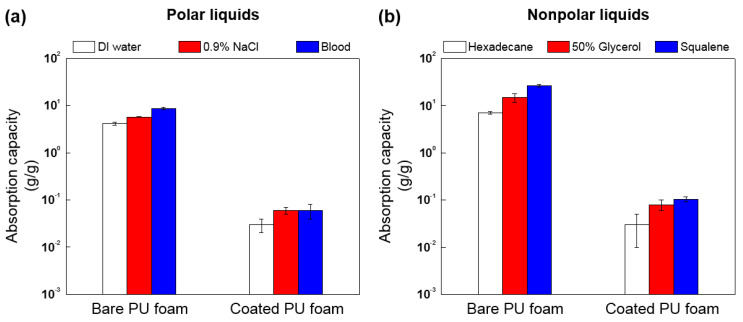
Absorption capacity of bare and FSCSNs-coated PU foams for (**a**) polar liquids (DI water, 0.9% NaCl, and blood) and (**b**) nonpolar liquids (hexadecane, 50% glycerol, and squalene).

An absorption test was conducted to confirm the ability of the fabricated PU foam to prevent contamination by polar and nonpolar liquids. The absorption capacities for the liquids were calculated using Equation (1), and the results are shown in Figure 8. As for the polar liquids, the average absorption capacity of DI water was 4.2 (g/g) for the bare PU foam and 0.1 (g/g) for the coated PU foam. The average absorption capacity of sodium chloride solution was 5.8 (g/g) for the bare PU foam and 0.1 (g/g) for the coated PU foam. Finally, the average absorption capacity of blood was the highest at 8.7 (g/g) for the bare PU foam and 0.1 (g/g) for the coated PU foam. From these results, the PU foam coated with FSCSNs showed a very high average reduction rate of the absorption capacity of polar liquids of more than 99%. Absorption capacity tests for nonpolar liquids also showed similar results. The average absorption capacity of hexadecane was 7.1 (g/g) for bare PU foam and 0.1 (g/g) for coated PU foam. The average absorption capacity of glycerol solution was 14.9 (g/g) for bare PU foam and 0.1 (g/g) for coated PU foam. Finally, the average absorption capacity of squalene was the highest at 26.5 (g/g) for the bare PU foam and at 0.1 (g/g) for the coated PU foam. In other words, PU foam coated with FSCSNs also showed an average absorption reduction rate of more than 99% for nonpolar liquids, confirming excellent water and oil absorption prevention capabilities. Therefore, as the elasticity of the PU foam did not decrease due to the absorption of liquid contaminants, it could be used as a mattress material for a long period of time [41,42]. 

The liquids used in the absorption experiment are all commonly found in medical facilities. Body fluid consists of 1% sodium chloride and various salts and organic components, and body sebum contains an average of 6% to 13% squalene, although there are differences according to gender and age [43,44]. Therefore, a mattress that comes in close contact with the body must have properties that prevent the absorption of such body fluids. In addition, glycerol is a low-toxicity substance that is used in the food, medicine, and cosmetic industries and is the most effective and widely used optical clearing agent among various types of alcohol [45,46]. According to a study by Lai et al., medical facilities use a 50% glycerol solution that is safe for skin application in vivo as a light scattering attenuator. Based on the literature, the concentration of glycerol was set to 50% for absorption experiments [47].

### 3.5. Mechanical Durability of FSCSNs-Coated PU Foam

**Figure 9:**
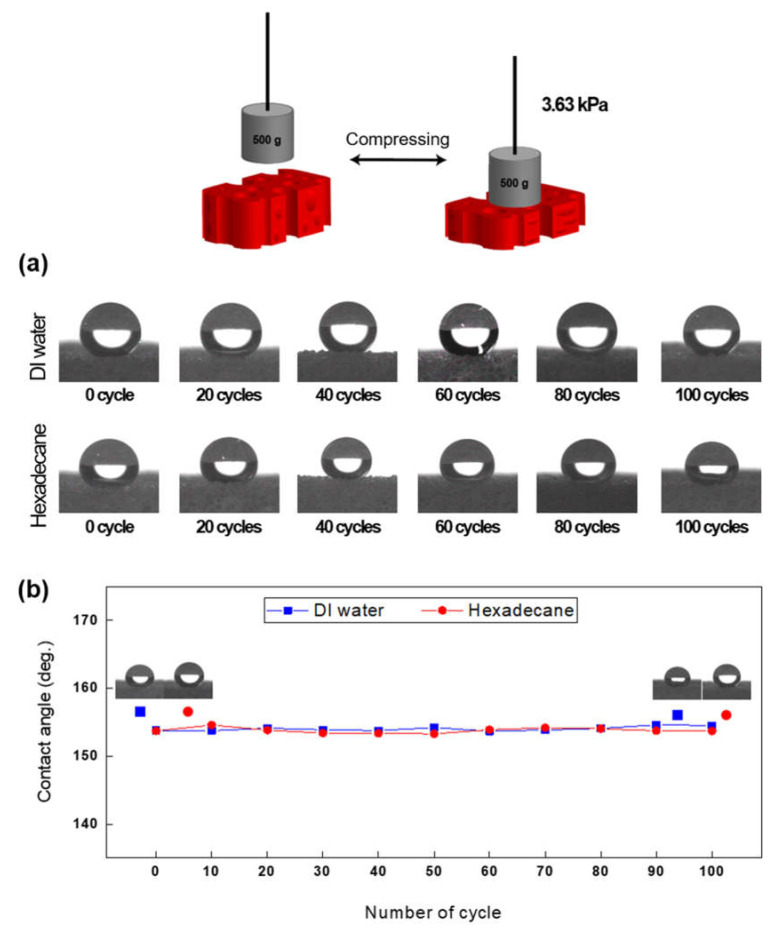
(**a**) Sequential photos showing the water contact angle and oil contact angle after multiple iterations of a compression test. (**b**) The contact angle measurements graph of FSCSNs-coated foam during the cyclic compression test.

Coated omniphobic PU foam is a mattress material, and due to its nature, it must maintain the omniphobic property of high water and oil repellency even under frequent pressure for a long period of time. Therefore, in this study, repeated compression tests were conducted to confirm the mechanical durability of the PU foam coated with FSCSNs. The weights and sizes of the samples used in the experiment were selected with reference to previous studies showing that the average interfacial pressure applied to a mattress by an adult male weighing 80 kg is 4 kPa to 8 kPa [48]. Based on this, the size of the PU foam coated with FSCSNs was set to 1.5 cm × 1.5 cm × 1.5 cm, and a pressure similar to that described in the literature was applied by placing a 500 g weight. As shown in Figure 9**,** to confirm whether the omniphobic properties were maintained after applying pressure for a certain number of times, the contact angles for water and hexadecane were measured every 20 cycles, and the results were summarized in a graph. The contact angles for water and hexadecane showed a high repellency of more than 150° throughout the experiment. The contact angle measured after applying pressure 100 times was 154.6° for DI water and 153.7° for hexadecane, confirming that the omniphobic properties were maintained. The porous ratio and depth present in polyurethane foam do not affect its resilience property [49]. Therefore, an omniphobic PU foam with a high proportion of an open-cell structure maintains its resilience related to mechanical durability. As a result, it retains the performance and omniphobic characteristics even under repeated compression.

### 3.6. Chemical Leaching Test

**Figure 10:**
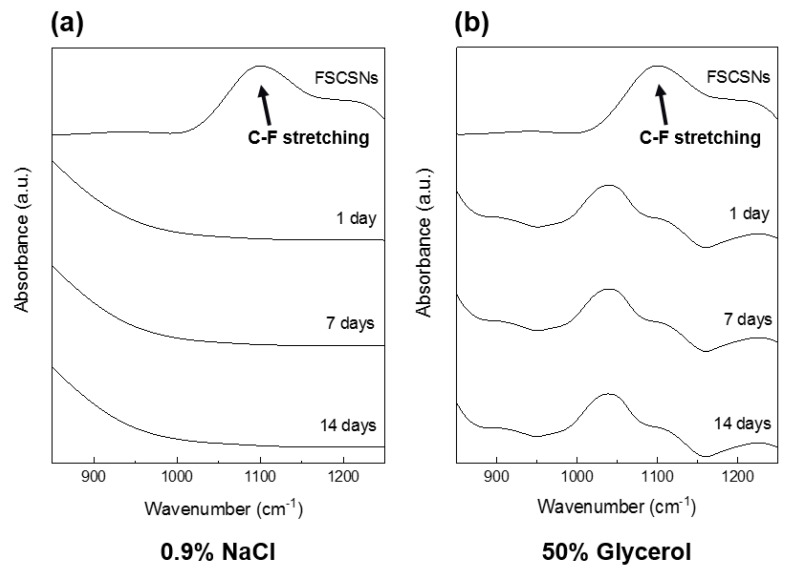
FTIR spectra of aliquots collected from samples of the FSCSNs-coated foam that had been immersed for up to 14 d in (**a**) 0.9% NaCl and (**b**) 50% glycerol, showing the absence of C−F stretching.

Because PU foam is used in an environment where there is frequent contact with the body, such as a mattress in a medical facility, the potential toxicity of the PU foam coating must be evaluated. Therefore, chemical leaching should not occur due to the separation or decomposition of fluorine groups on the surface of silica nanoparticles functionalized with HFTCS. Therefore, in this study, leaching experiments were conducted using FTIR for two types of liquids to confirm the chemical stability of the PU foam coated with FSCSNs (Figure 10). The graph in Figure 10a shows the results of leaching after immersing the coated PU foam in a 0.9% sodium chloride solution, a polar liquid often used in medical facilities, for up to 14 d. When compared with the FSCSN suspension as the control group, unbound FSCSN molecules exhibited a C−F stretching peak around 1050 cm^−1^. However, no peak was observed for at least 14 d in the 0.9% sodium chloride solution in which the coated PU foam was immersed. When a saline solution containing Na and Cl ions is present, each ion causes a specific arrangement of water molecules, impacting the OH stretching band, which is primarily observed around 3300 cm^−1^. Consequently, no peak is observable for the 0.9% NaCl solution within the measured wavelength range of 850 cm^−1^ to 1250 cm^−1^ [50].

The graph in Figure 10b shows the results of an experiment confirming chemical stability using a 50% glycerol solution, which is a nonpolar liquid. Except for the C−O−C stretching peak of the 50% glycerol solution around 1103 cm^−1^, no peaks were observed, including a C−F stretching peak around 1050 cm^−1^. This means that no chemical leaching occurred in the 50% glycerol solution in which the PU foam coated with FSCSNs was immersed [51]. Furthermore, glycerol-containing water exhibits primary and secondary hydroxyl groups within the range of 1000−1100 cm^−1^, leading to observable peaks at those specific wavelengths [52]. Moreover, the C−F stretching shows a strong peak at 1050 cm^−1^, attributed to the overlap between the Si−O−Si bond and the −CF_3_ symmetric stretching. However, the influence of NaCl and glycerol on the C−F stretching is minimal, as no significant changes were observed [53]. No chemicals were detected within the detection limit of 1 ppm for at least 14 d in either liquid used, indicating that the fabricated PU foam is chemically stable in both polar and nonpolar liquids.

## 4. Conclusions

To solve the problem of cross-contamination, which has become more important due to the emergence of infectious diseases, this study aimed to prevent the contamination of mattresses that come in close contact with the human body in medical facilities. The surface of PU foam, the main material used in such mattresses, was coated with silica nanoparticles functionalized with fluorinated silane to reduce surface energy and form nanoscale surface roughness. The key results were the following: (1) The treated PU foam exhibited high repellency to liquids such as water and oil; (2) the adhesion of harmful bacteria such as *E. coli* O157:H7 and *S. epidermidis* was reduced by more than 90% per unit area (1–2 log units) compared to untreated PU foam; and (3) a high antifouling effect against polar and nonpolar liquid pollutants common in medical facilities was observed. The durability of the prepared PU foam was confirmed through repeated compression experiments. The experiment was conducted considering the average interfacial pressure applied to the mattress by adults, and as a result, the omniphobic properties were maintained even after repeated compression up to 100 times. Furthermore, to confirm the chemical stability, leaching experiments were conducted under the conditions of a 0.9% sodium chloride solution and a 50% glycerol solution. Peaks corresponding to carbon and fluorine bonds could not be identified in the two solutions for up to 2 weeks. Based on the findings of this study, the PU foam coated with FSCSNs has a high antifouling effect due to water and oil repellency as well as anti-adhesion properties against bacteria. Moreover, the proposed coating method is simple and scalable and employs inexpensive reagents. In addition to the medical field, it is expected to be applicable to various industries that require hygiene, such as food, electronics, and textiles.

## Data Availability

The data that support the findings of this study are available upon request from the authors.
